# Supplementary material for: Efficient intersystem crossing and tunable ultralong organic room-temperature phosphorescence via doping polyvinylpyrrolidone with polyaromatic hydrocarbons
Source: Nat Commun. 2024 Jun 1;15:4674. doi: 10.1038/s41467-024-48913-x (PMC11144212; doi:10.1038/s41467-024-48913-x)
Supplement: Supplementary file 1 — Supplementary Information [file 41467_2024_48913_MOESM1_ESM.pdf]

Supplementary Information

Efficient intersystem crossing and tunable ultralong organic room-temperature phosphorescence via doping polyvinylpyrrolidone with polyaromatic hydrocarbons

Guangxin Yang[1], Subin Hao[1], Xin Deng[1], Xinluo Song[1], Bo Sun[*2], Woo Jin Hyun[3], Ming-De Li[*1,4], Li Dang[*1]

[1] College of Chemistry and Chemical Engineering, Key (Guangdong-Hong Kong Joint) Laboratory for Preparation and Application of Ordered Structural Materials of Guangdong Province, Shantou University, Guangdong 515063, P. R. China.

[2] State & Local Joint Engineering Research Center for Ecological Treatment Technology of Urban Water Pollution, College of Life and Environmental Science, Institute for Eco-environmental Research of Sanyang Wetland, Wenzhou University, Wenzhou, Zhejiang 325035, PR China.

[3] Department of Materials Science and Engineering, Guangdong Technion-Israel Institute of Technology, Shantou, Guangdong 515063, China.

[4] Chemistry and Chemical Engineering Guangdong Laboratory, Shantou 515031, China.

[*] Correspondence to: E-mail: sunbo@wzu.edu.cn;ldang@stu.edu.cn; mdli@stu.edu.cn

Table of Contents

Supplementary Figure 1. The steady-state photoluminescence spectrum of films upon 365 nm excitation............3

Supplementary Figure 2. Fluorescence and phosphorescence images of PVP film under UV light at 365 nm........3

Supplementary Figure 3. Delayed emission spectra of the guest compounds in 2-MeTHF (10 μM) at 77 K...........4

Supplementary Figure 4. Variation of the delayed emission spectrum of BeCPh/PVP and BePh/PVP (1 ms delayed, annealing temperature :80 °C)..4

Supplementary Figure 5. The UV-Vis spectrum of PVP film (left), The UV-Vis spectrum of BecPh, BePh, FlAn, Py, BeAn, Pi, BeTe,DBeCh,BePe and Co powders (right)..5

Supplementary Figure 6. The fs-TA for Py (a) ($c=10^{-5}$ mol/l) and (b) ($c=10^{-2}$ mol/l) recorded in MeCN solution after 365 nm excitation...5

Supplementary Figure 7. Shown are fs-TA spectra of Py/PVP (a) and BePe/PVP (b) films at the beginning delay times after the activation with 365 nm UV light, respectively...5

Supplementary Figure 8. The fs-TA spectra of Py/PVP film after 365 nm excitation...5

Supplementary Figure 9. The fs-TA spectra decay curves in 370 nm of Py/PMMA and Py/PVP film after 365 nm excitation...6

Supplementary Figure 10. The fs-TA for BePe recorded in DCM solution after 365 nm excitation.......................6

Supplementary Figure 11. (a) and (c) serve as blank controls for the mapping image of fs-TA spectra of BecPh/PMMA and DBeCh/PMMA doped films, the mapping image of fs-TA spectra of BecPh/PVP (b) and DBeCh/PVP (d) film after activation with 365 nm UV light, respectively...6

Supplementary Figure 12. The HOMOs and LUMOs of VP,$(VP)_2$,$(VP)_5$,$(VP)_{10}$,BecPh,BePh,FlAn,Py, BeAn,Pi,BeTe,DBeCh,BePe and Co...7

Supplementary Figure 13. (a) The fs-TA spectra of Py with different concentrations in MeCN after 320 nm excitation. (b) The fs-TA spectra of BePe with different concentrations in DCM after 365 nm excitation.................8

Supplementary Figure 14. Phosphorescence images of PMMA and PVP films doped with the same mass ratio of Py under UV light at 365 nm...8

Supplementary Table 1. Energies of S_1, T_1, T_2, Energy Gaps (in eV) between S_1 and T_1/T_2 States and SOC (spin orbit coupling constants) of VP, VP-BecPh, VP-BePh, VP-FlAn, VP-Py, VP-BeAn, VP-Pi, VP-BeTe, VP-DBeCh, VP-BePe and VP-Co...9

Supplementary Figure 15. Luminescent images of Py/PVP and FlAn/PVP as coatings under Daylight, on UV lamp (365 nm) excitation,and after removal of UV light..9

Supplementary Figure 16. (a)Switching of the phosphorescence intensity of BecPh/PVP upon photoactivation and thermal annealing. (b)Switching of the delayed emission spectra (1 ms delayed) of BecPh/PVP upon photoactivation and thermalannealing at the first and fifth cycles (annealing temperature :80 °C)..........................10

Supplementary Figure 17. Luminescent images of programmable label based on Py/PVP (bottom) and FlAn/PVP (above) film attached to quartz flakes...10

Supplementary Figure 18. Luminescent images of programmable label of Py/PVP and FlAn/PVP solution coated on paper...10

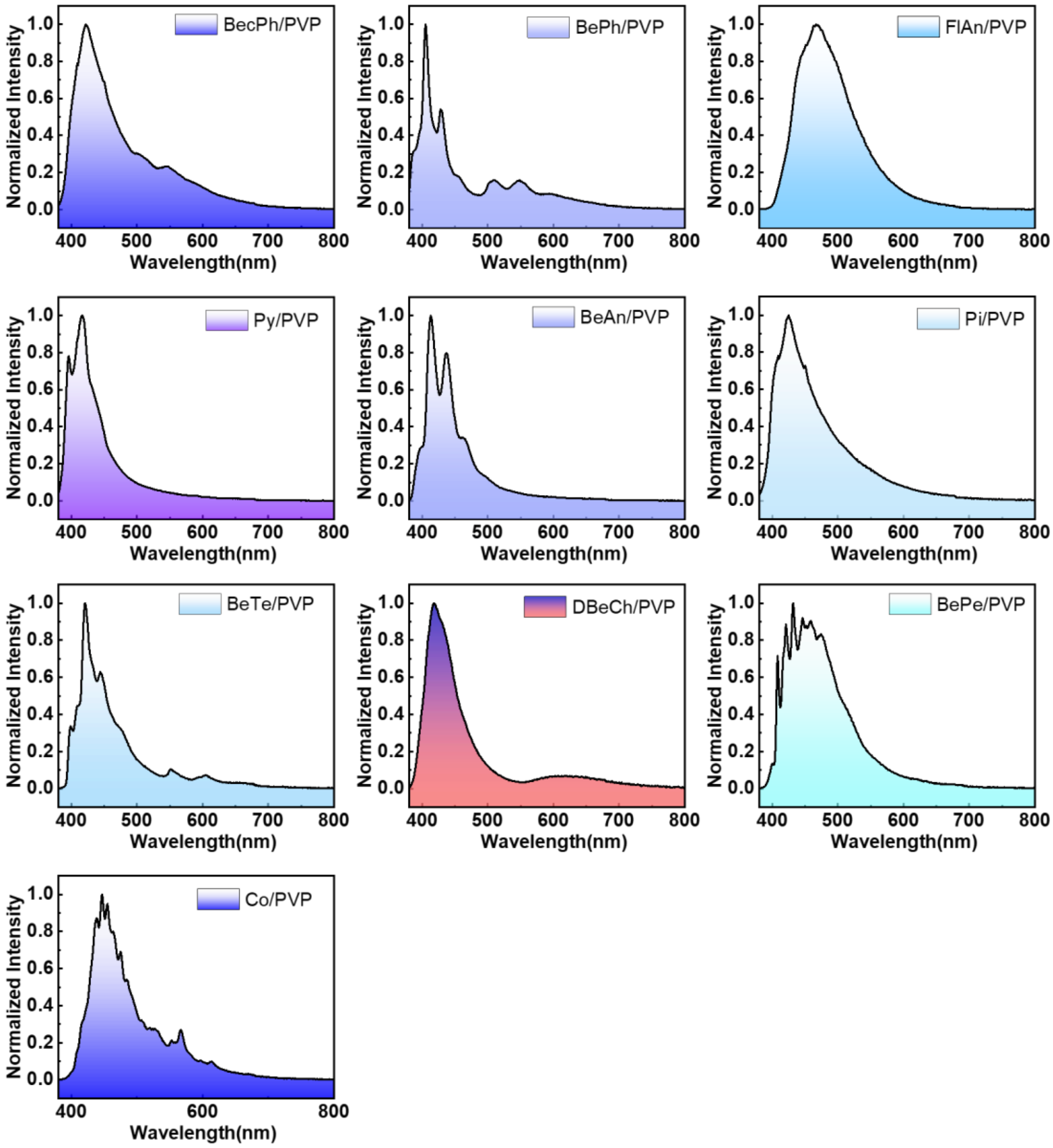

Supplementary Figure 1. The steady-state photoluminescence spectrum of BecPh/PVP, BePh/PVP, FlAn/PVP, Py/PVP, BeAn/PVP, Pi/PVP, BeTe/PVP, DBeCh/PVP, BePe/PVP and Co/PVP films upon 365 nm excitation.

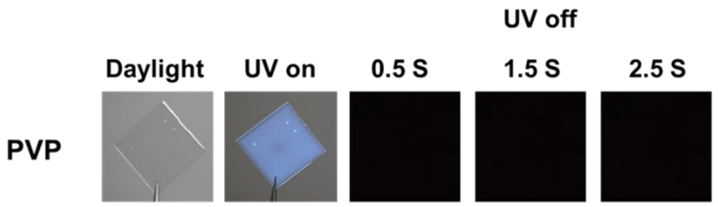

Supplementary Figure 2. Fluorescence and phosphorescence images of PVP film under UV light at 365 nm.

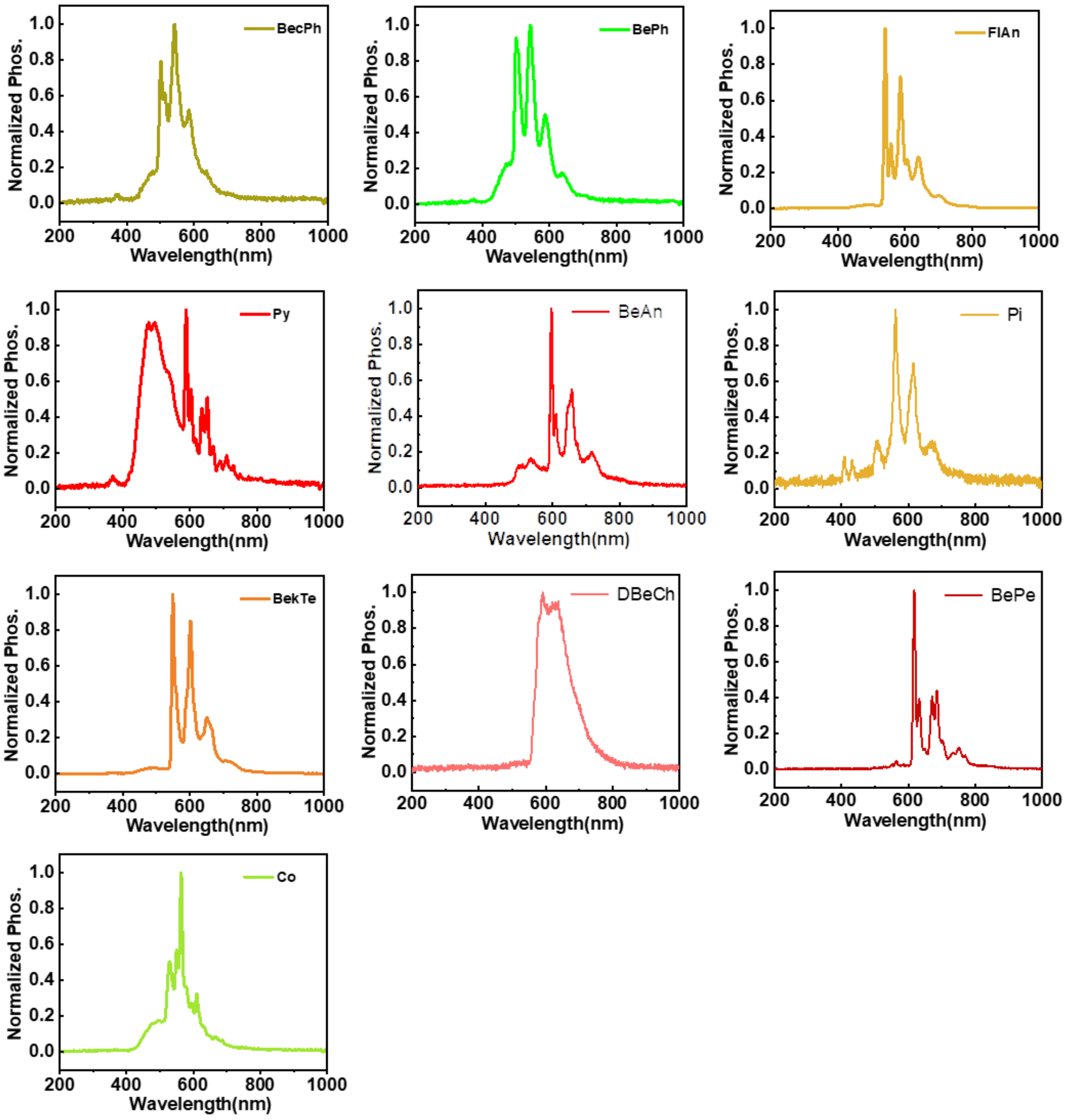

Supplementary Figure 3. Delayed emission spectra of the guest compounds in 2-MeTHF (10 μM) at 77 K.

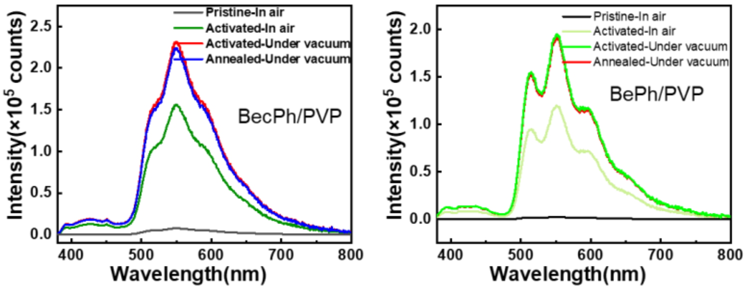

Supplementary Figure 4. Variation of the delayed emission spectrum of BeCPh/PVP and BePh/PVP (1 ms delayed, annealing temperature :80 °C)

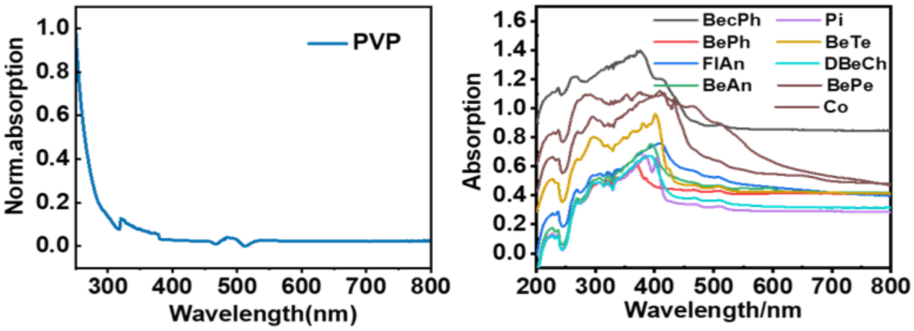

Supplementary Figure 5. The UV-Vis spectrum of PVP film (left), The UV-Vis spectrum of BecPh, BePh, FlAn, Py, BeAn, Pi, BeTe, DBeCh, BePe and Co powders (right).

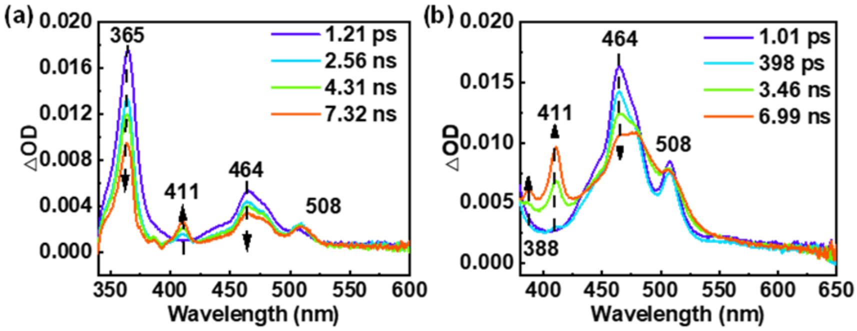

Supplementary Figure 6. The fs-TA for Py (a) ($c=10^{-5}$ mol/l) and (b) ($c=10^{-2}$ mol/l) were recorded in MeCN solution after 365 nm excitation.

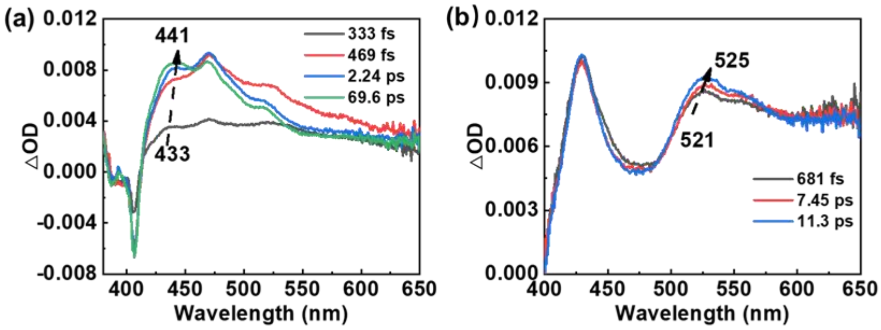

Supplementary Figure 7. Shown are fs-TA spectra of Py/PVP (a) and BePe/PVP (b) films at early delay times after the activation with 365 nm.

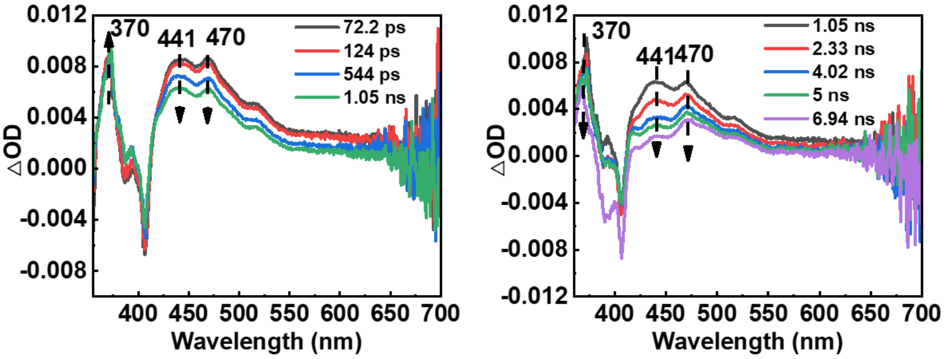

Supplementary Figure 8. The fs-TA spectra of Py/PVP film after 365 nm excitation.

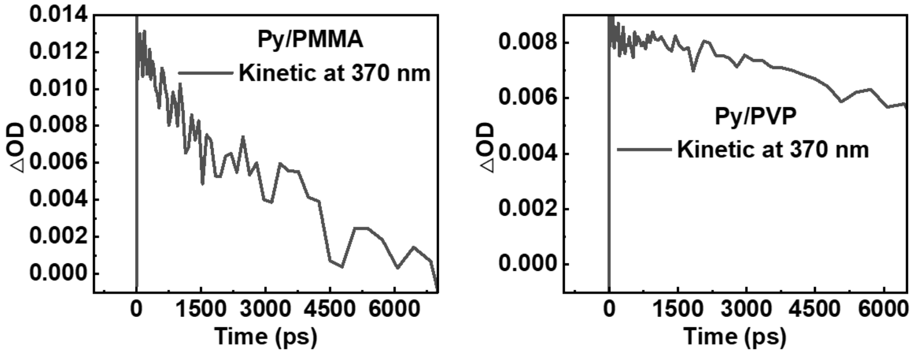

Supplementary Figure 9. The fs-TA spectra decay curves at 370 nm of Py/PMMA and Py/PVP films after 365 nm excitation.

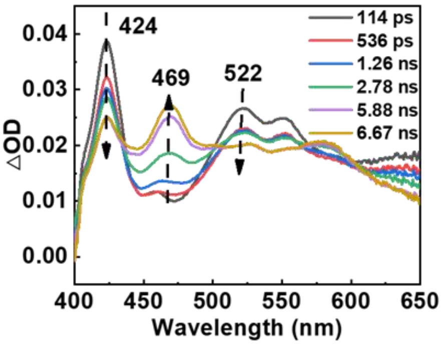

Supplementary Figure 10. The fs-TA for BePe (c=10^{-2} mol/L) was recorded in DCM solution after 365 nm excitation.

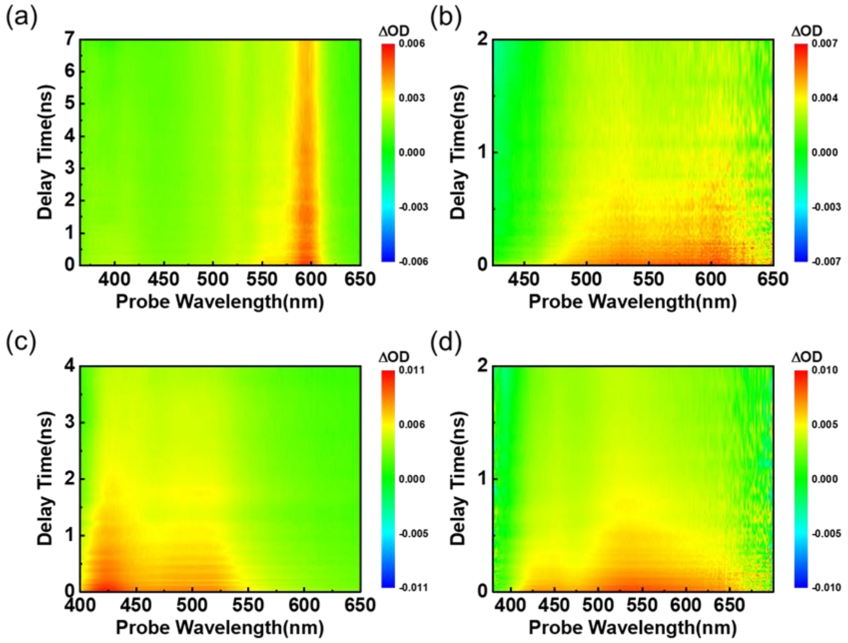

Supplementary Figure 11. (a) and (c) serve as blank controls for the mapping image of fs-TA spectra of BecPh/PMMA and DBeCh/PMMA doped films, the mapping image of fs-TA spectra of BecPh/PVP (b) and DBeCh/PVP (d) films after activation with 365 nm UV light, respectively.

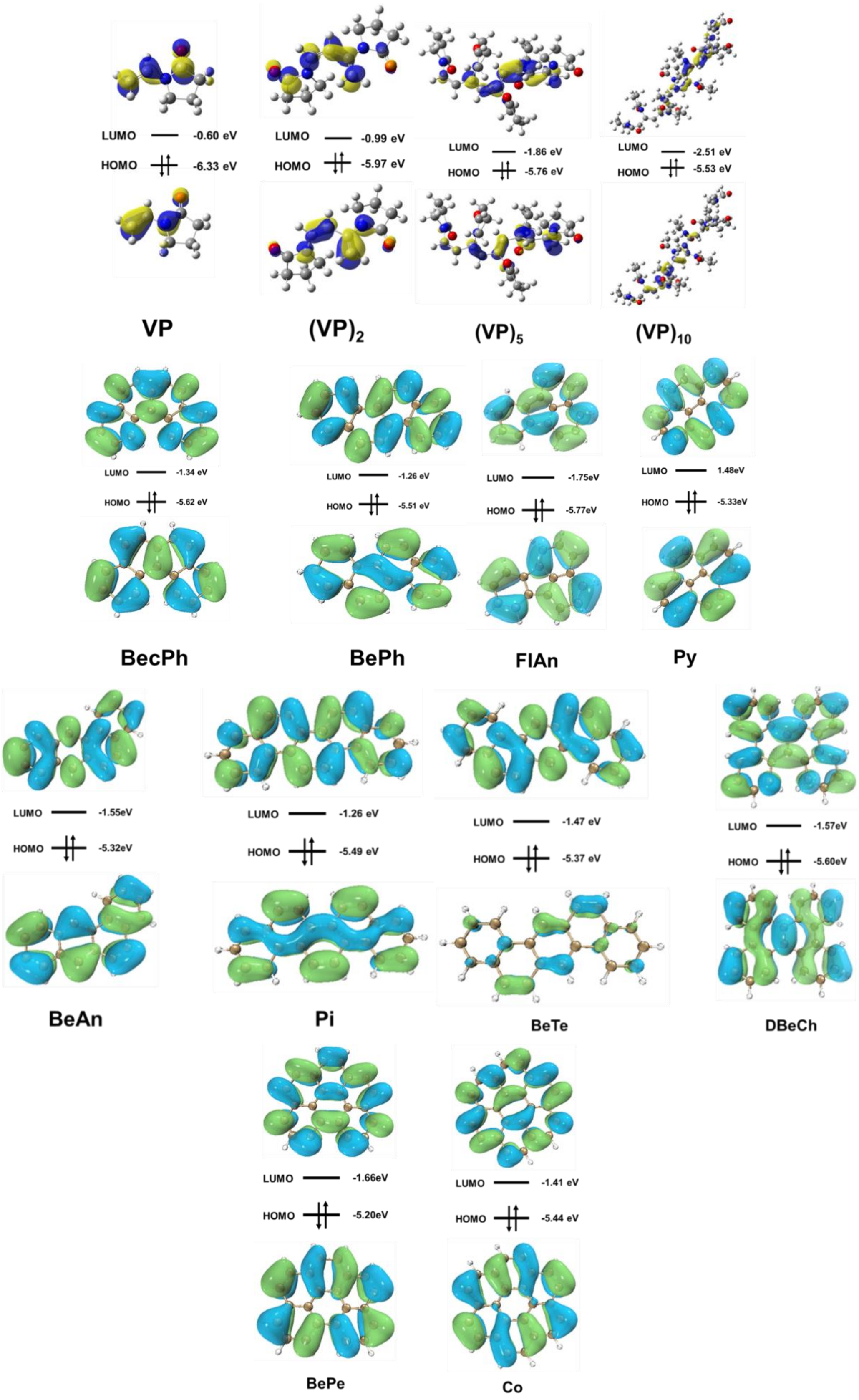

Supplementary Figure 12. The HOMOs and LUMOs of VP, $(VP)_2$, $(VP)_5$, $(VP)_{10}$, BecPh, BePh, FlAn, Py, BeAn, Pi, BeTe, DBeCh, BePe and Co.

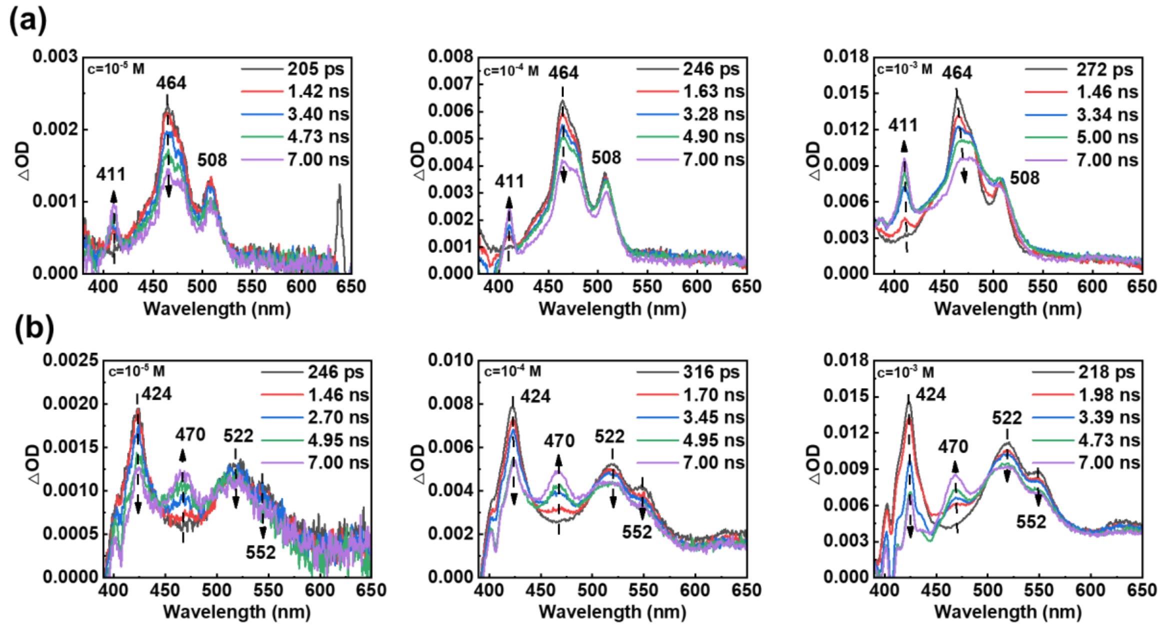

Supplementary Figure 13. (a) The fs-TA spectra of Py with different concentrations in MeCN after 320 nm excitation. (b) The fs-TA spectra of BePe with different concentrations in DCM after 365 nm excitation.

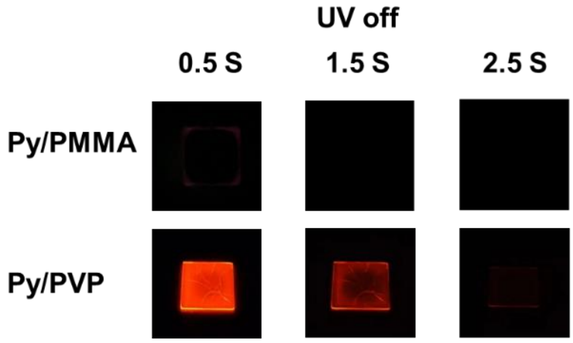

Supplementary Figure 14. Phosphorescence images of PMMA and PVP films doped with the same mass ratio of Py under UV light at 365 nm.

Supplementary Table 1. Energies of S_1, T_1, T_2, Energy Gaps (in eV) between S_1 and T_1/T_2 States and SOC (spin orbit coupling constants) of VP, VP-BecPh, VP-BePh, VP-FlAn, VP-Py, VP-BeAn, VP-Pi, VP-BeTe, VP-DBeCh, VP-BePe and VP-Co (B3LYP/6-31G* and TD-B3LYP/6-31G*).

Sample	S_1 (eV)	T_1 (eV)	T_2 (eV)	$\Delta E_{(S1-T1)}$ (eV)	$\Delta E_{(S1-T2)}$ (eV)	$SOC_{(S1-T1)}$	$SOC_{(S1-T2)}$
VP	5.05	3.48	4.60	1.57	0.45	13.883	1.783
VP-BecPh	3.598	2.486	3.108	1.11	0.49	0.118	0.606
VP-BePh	3.588	2.507	3.25	1.08	0.34	0.153	0.061
VP-FlAn	3.119	2.382	2.618	0.74	0.50	0.162	0.085
VP-Py	3.328	2.127	3.241	1.20	0.09	0.203	0.320
VP-BeAn	3.39	2.07	3.06	1.32	0.34	0.014	0.083
VP-Pi	3.596	2.49	2.964	1.11	0.63	0.063	0.082
VP-BeTe	3.438	2.265	3.099	1.17	0.34	0.028	0.065
VP-DBeCh	3.467	2.28	3.236	1.19	0.23	0.211	0.717
VP-BePe	3.208	2.048	3.037	1.16	0.17	0.146	0.099
VP-Co	3.233	2.346	2.969	0.89	0.26	0.030	0.033

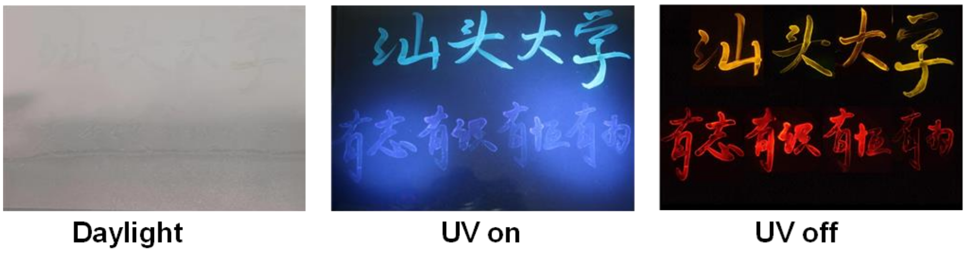

Supplementary Figure 15. Luminescent images of Py/PVP and FlAn/PVP as coatings under Daylight, on UV lamp (365 nm) excitation, and after removal of UV light.

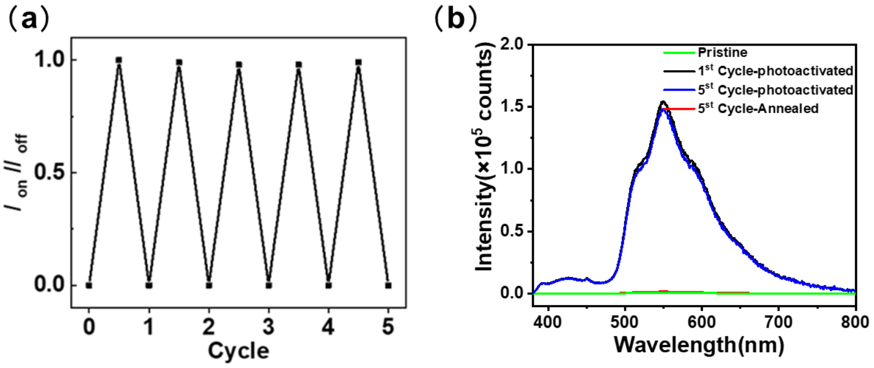

Supplementary Figure 16. (a) Switching of the phosphorescence intensity of BecPh/PVP upon photoactivation and thermal annealing. I_{on} represents the phosphorescence intensity at 550 nm after photoactivation, while I_{off} is the one after thermal annealing. (b) Switching off the delayed emission spectra (1 ms delayed) of BecPh/PVP upon photoactivation and thermal annealing at the first and fifth cycles(annealing temperature:80 °C).

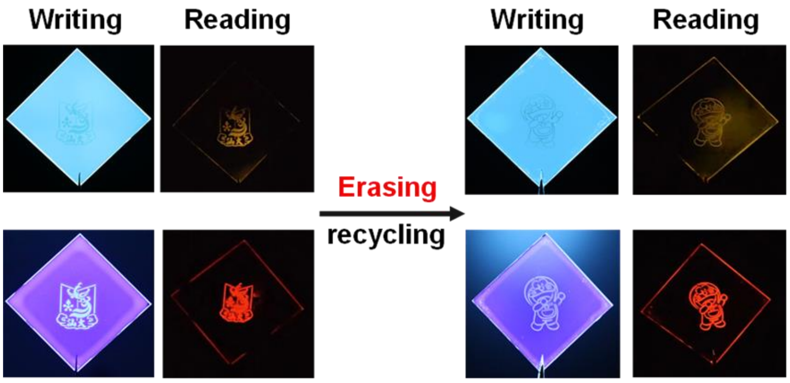

Supplementary Figure 17. Luminescent images of programmable labels based on Py/PVP (bottom) and FlAn/PVP (above) film attached to quartz flakes.

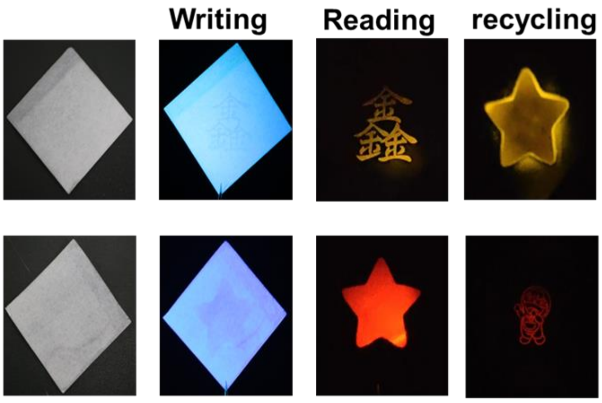

Supplementary Figure 18. Luminescent images of programmable labels of Py/PVP and FlAn/PVP solution coated on paper.
