# Supplementary material for: Efficient intersystem crossing and tunable ultralong organic room-temperature phosphorescence via doping polyvinylpyrrolidone with polyaromatic hydrocarbons
Source: Nat Commun. 2024 Jun 1;15:4674. doi: 10.1038/s41467-024-48913-x (PMC11144212; doi:10.1038/s41467-024-48913-x)
Supplement: Supplementary file 3 — Description of Additional Supplementary Files [file 41467_2024_48913_MOESM3_ESM.pdf]

Description of Additional Supplementary Files

File Name: Supplementary Data 1
Description: Cartesian coordinates for the complexes (VP, (VP)2, (VP)5, (VP)10, BecPh, BePh, FlAn, Py, BeAn, Pi, BeTe, DBeCh, BePe, Co) calculated in the study.

File Name: Supplementary Movie 1
Description: The movie of room temperature phosphorescence of DbeCh-PVP.

File Name: Supplementary Movie 2
Description: The movie of room temperature phosphorescence of BeTe-PVP.

File Name: Supplementary Movie 3
Description: The movie of room temperature phosphorescence of Pi-PVP.

File Name: Supplementary Movie 4
Description: The movie of room temperature phosphorescence of BeAn-PVP.

File Name: Supplementary Movie 5
Description: The movie of room temperature phosphorescence of Py-PVP.

File Name: Supplementary Movie 6
Description: The movie of room temperature phosphorescence of FlAn-PVP.

File Name: Supplementary Movie 7
Description: The movie of room temperature phosphorescence of BePh-PVP.

File Name: Supplementary Movie 8
Description: The movie of room temperature phosphorescence of BecPh-PVP.

File Name: Supplementary Movie 9
Description: The movie of room temperature phosphorescence of BePe-PVP.

File Name: Supplementary Movie 10
Description: The movie of room temperature phosphorescence of Co-PVP.
